# Cough-Induced Rib Fracture in a Postmenopausal Woman Without Clinical Evidence of Osteoporosis: A Case Report

**DOI:** 10.7759/cureus.75383

**Published:** 2024-12-09

**Authors:** Omar Namiq, Namir G Al-Tawil

**Affiliations:** 1 College of Medicine, Hawler Medical University, Erbil, IRQ; 2 Department of Community Medicine, College of Medicine, Hawler Medical University, Erbil, IRQ

**Keywords:** chest pain, cough, cough-induced rib fracture, postmenopausal woman, rib fracture

## Abstract

Cough-induced rib fractures are rare conditions and are seldom reported in the medical literature. This case involves a 54-year-old postmenopausal woman who experienced a persistent dry cough lasting 16 days, which progressed to acute, localized chest pain in the right hemithorax. Symptoms started after an initial chest infection. She developed a fracture of her right fifth rib due to a cough, a rare but serious complication of hard work by the cough. The patient achieved a favorable outcome through conservative management, which remains the preferred initial approach for cough-induced rib fractures. The patient's clinical presentation, diagnostic findings, and treatment plan are presented herein. The case, therefore, overemphasizes the consideration of rib fractures in patients presenting with protracted or severe coughing with chest pain.

## Introduction

Coughing is a natural instinctive reflex that serves as a defense mechanism within the immune system, helping clear foreign substances from the respiratory tract [1]. Cough-induced rib fractures occur when repetitive mechanical stress from intense coughing exceeds the ribs' capacity to withstand force, leading to fractures at their weakest points [2]. Rib fractures from a cough are less common but a recognized complication of chronic or violent coughing fits. Most cough-induced rib fractures occur in acute upper respiratory tract infection, chronic obstructive pulmonary disease, and pneumonia [3]. Rib fractures are usually traumatic [4], but they can occur as a result of non-traumatic causes like severe coughing, especially in patients with underlying conditions that weaken the skeletal structure, such as osteoporosis [5]. Fractures can occur even without traditional risk factors, including in younger patients during periods of extreme coughing that stress the ribs [6]. This case highlights a rare instance of cough-induced rib fracture in a postmenopausal woman without clinical evidence of osteoporosis, illustrating the need for clinicians to consider such diagnoses in cases of localized pain after a cough.

## Case presentation

A 54-year-old postmenopausal female housewife from Erbil presented to the hospital with a 16-day history of persistent dry cough, followed by sudden, severe chest pain, prompting her to seek medical attention. Her symptoms initially began with an upper respiratory tract infection, for which she was treated with amoxicillin. While fever and body aches improved, the dry cough and intermittent sweating persisted. The patient reported episodes of severe nocturnal coughing, productive of thick, pale yellow sputum.

A few days before the presentation, she developed sudden, stabbing pain localized to the right side of the chest, below the armpit, radiating to the back. The pain intensified with deep inspiration and coughing but was partially relieved with muscle relaxants. Clinical examination revealed normal air entry with no added sounds.

The patient was a non-smoker but had been exposed to secondhand smoke from her husband. She denied any past medical history of osteoporosis or gastrointestinal, cardiovascular, or neurological conditions. Despite the increased risk of osteoporosis in postmenopausal women, she reported no personal or family history of bone-related issues.

**Figure 1:**
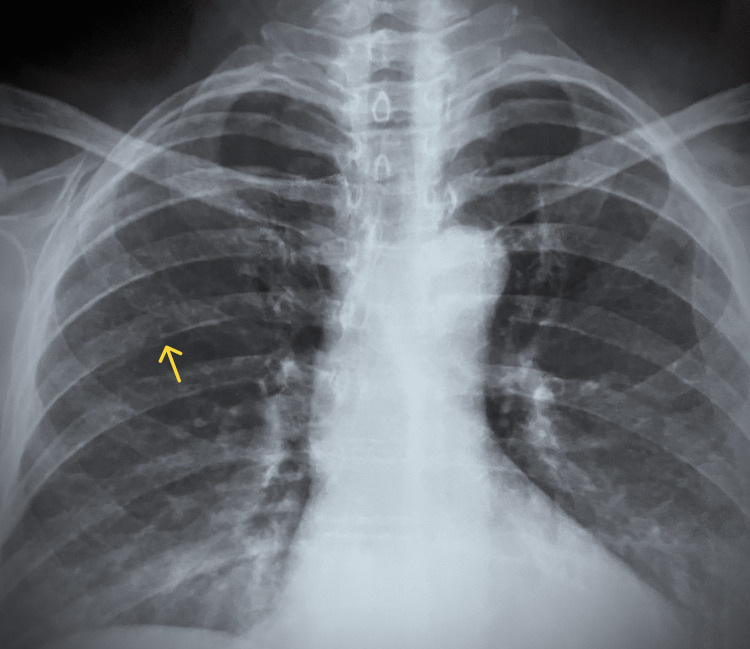
Chest X-ray of the patient showing clear lung fields with a rib fracture indicated by a yellow arrow on the right side. The cardiothoracic ratio and hila appear normal.

Laboratory investigations showed a normal complete blood count and C-reactive protein (CRP), ruling out significant infection or ongoing inflammation (Table 1). A chest X-ray revealed clear lung fields, a normal cardiothoracic ratio, and normal hila, indicating no evidence of lung or cardiovascular abnormalities. However, imaging confirmed the presence of a right-sided rib fracture. No additional skeletal deformities or pathological lesions were observed (Figure 1).

**Table 1:** Laboratory results of the patient to assess underlying causes of rib fracture.

Parameter	Result	Laboratory Reference Range
CBC		
Hemoglobin (Hb)	12.6 g/dL	13.8–17.2 g/dL (Men), 12.1–15.1 g/dL (Women)
Hematocrit (Hct)	38%	38.8–50% (Men), 34.9–44.5% (Women)
Red Blood Cell Count (RBC)	4.7 million/µL	4.7–6.1 million/µL (Men), 4.2–5.4 million/µL (Women)
White Blood Cell Count (WBC)	7,000/µL	4,000–11,000/µL
Platelet Count	220,000/µL	150,000–450,000/µL
Mean Corpuscular Volume (MCV)	92 fL	80–100 fL
Mean Corpuscular Hemoglobin (MCH)	28 pg	27–33 pg
Mean Corpuscular Hemoglobin Concentration (MCHC)	33 g/dL	32–36 g/dL
C-Reactive Protein	<1 mg/L	<5 mg/L
Erythrocyte Sedimentation Rate (ESR)	14 mm/hour	< 20 mm/hour
Calcium (Corrected)	9.1 mg/dL	8.5-10.5 mg/dL
Phosphate	3.8 mg/dL	2.5-4.5 mg/dL
Vitamin D	32 ng/mL	30-50 ng/mL (Sufficient)
Alkaline Phosphatase (ALP)	132 U/L	44-147 U/L
Thyroid-stimulating hormone (TSH)	3.9 μIU/mL	0.5-4.5 μIU/mL

Blood calcium measurements and other relevant laboratory studies, including phosphate, vitamin D, and alkaline phosphatase levels, were within normal ranges, ruling out metabolic or endocrine abnormalities as secondary etiologies of the pathological rib fracture. Additionally, inflammatory markers such as CRP and erythrocyte sedimentation rate (ESR) were unremarkable, further supporting the absence of underlying systemic disease (Table 1).

Treatment involved the use of analgesics (nonsteroidal anti-inflammatory drugs), topical analgesics, and cough syrup to control symptoms. The patient was advised to avoid vigorous physical activity and was followed up after two weeks. At the follow-up visit, the patient reported full resolution of symptoms, including chest pain and cough, and was able to resume her daily activities.

## Discussion

While rare, cough-induced rib fractures can occur due to prolonged or severe coughing episodes, even in individuals without underlying bone conditions [3]. Although postmenopausal, this patient denied any history of osteoporosis, suggesting that her rib fracture was a consequence of the mechanical stress from coughing.

Osteoporosis is a common condition in postmenopausal women, and it can increase the risk of fractures from minimal force [7]. Although osteoporosis was not formally diagnosed in this patient, her postmenopausal status still placed her at a higher risk for bone weakening, which may have contributed to the rib fracture. No clinical signs or prior fractures suggestive of osteoporosis were present, although the condition was not definitively ruled out.

Cough-induced rib fractures can be misdiagnosed or underdiagnosed due to their non-specific presentation. Patients typically present with chest pain exacerbated by movement, deep breathing, or further coughing episodes, which may be mistaken for musculoskeletal pain, pneumonia, or pneumothorax [6]. In this case, the diagnosis was confirmed with a chest X-ray, which is essential for differentiating between other causes of chest pain and rib fractures.

The management of cough-induced rib fractures primarily focuses on treating the underlying cause of the cough and providing adequate pain relief [8]. In this patient, conservative management with analgesics, topical pain relievers, and cough suppression was successful, and her symptoms fully resolved within two weeks.

This case highlights the importance of maintaining a high index of suspicion for rib fractures in patients with persistent cough and chest pain, especially when the chest pain is localized, worsens with movement, and is associated with deep breaths or further coughing. It also underscores the value of imaging studies, such as chest X-rays, in confirming the diagnosis and guiding management.

A significant limitation of this case is that the patient refused to undergo a DEXA (dual-energy X-ray absorptiometry) scan, which limited the ability to exclude osteoporosis definitively. While clinical and other diagnostic findings were considered, the diagnosis of osteoporosis could not be entirely ruled out. Further investigation, particularly with imaging, may be necessary to assess bone density and fully rule out osteoporosis.

## Conclusions

Rib fractures due to coughing are a less common but clinically important cause of chest pain among patients with chronic or forceful cough. This case reminds healthcare providers that such a diagnosis must be considered even when traditional risk factors, including osteoporosis, are lacking. Appropriate early recognition and management will avoid misdiagnosis, ensure prompt treatment, and lead to rapid recovery. Clinicians, therefore, should be attentive to chest pain from chronic cough when they cannot identify its causes.

## Appendices

The perspective of the patient

Being diagnosed with a fractured rib due to a cough, I immediately thought the worst: that cancerous cells might have invaded my chest wall. Of course, this did not put my mind at ease, at least until my physician had reassured me regarding the condition of my disease. His explanation and support were truly comforting. After starting the treatment, I was glad to find that the pain would disappear gradually. Whereas the pain prevailed and highly affected my daily life and household duties, now I am feeling very different. I can resume my daily routine and manage my house as per my choice and without any hindrance. On the whole, I am highly satisfied with the care provided and thankful for the satisfactory outcome.
